# Supplementary material for: Exploratory Study on Application of MALDI-TOF-MS to Detect SARS-CoV-2 Infection in Human Saliva
Source: J Clin Med. 2022 Jan 6;11(2):295. doi: 10.3390/jcm11020295 (PMC8781148; doi:10.3390/jcm11020295)
Supplement: Supplementary file 1 [file jcm-11-00295-s001.zip › jcm-1489148-supplementary.pdf]

Supplementary Materials

Table S1. List of peaks distinguishing SARS-CoV-2 positive from negative samples after the application of Wilcoxon rank sum test (p<0.05).

Peaks (m/z)	p-value	**Ratio
2047.9	7.9e-04	31.1
*2102.4	1.9e-04	-41.4
2166.6	4.5e-02	16.0
2202.9	1.5e-03	-34.1
*2489.2	2.1e-07	81.3
2520.3	1.8e-02	14.9
2580.6	1.7e-02	14.0
2597	3.9e-02	-33.3
2687.7	4.7e-02	-27.7
2938.5	2.7e-03	52.7
*2977.2	9.1e-05	53.9
3203.5	4.8e-02	32.1
3220.3	1.0e-02	24.2
3494.4	5.6e-03	17.0
*3522.3	2.5e-05	60.1
3980.6	1.3e-03	35.6
4033.5	1.2e-03	80.9
4062.2	2.7e-02	50.8
4129.9	1.4e-02	-35.1
4191	3.2e-02	33.0
4394.1	8.1e-03	30.5
4449.2	3.5e-03	-45.1
4612.1	1.5e-02	30.4
4658.9	2.5e-02	16.9
4964.9	3.1e-02	41.9
5034.6	3.8e-03	92.7
*5063.3	3.2e-05	40.9
5099.3	3.7e-02	17.9
5134.5	2.5e-02	20.4
5153.9	1.1e-03	22.2
5233.9	9.9e-03	57.2
5380.4	1.3e-03	-38.3
*5418.9	1.4e-05	-58.5
*5463.3	3.3e-04	23.4
5503	4.9e-02	16.1
*5613.4	8.7e-05	37.7
5652.5	3.8e-03	8.8
5734.4	1.2e-02	22.3
*5814	3.0e-04	28.0
5867.2	1.3e-03	39.0
6305.7	1.3e-02	18.6
7376	1.4e-02	44.1
7647.3	4.5e-02	-37.9
7684.5	2.9e-03	-42.1
7853.8	3.0e-02	28.2
8145.2	5.9e-04	28.2
8300.7	1.3e-02	31.3
8741.5	3.1e-02	-30.1
9059.1	2.7e-02	35.4
9798.3	8.0e-03	60.3
9959.3	7.7e-03	-30.9
11004.3	2.7e-02	24.1

*Peaks identified with a two-tailed Wilcoxon rank sum test (p < 0.05) with a Benjamini-Hochberg correction (p < 0.1).

**Ratio computed from normalised mean intensities peaks between Cov- and Cov+ groups for the D0 time point.

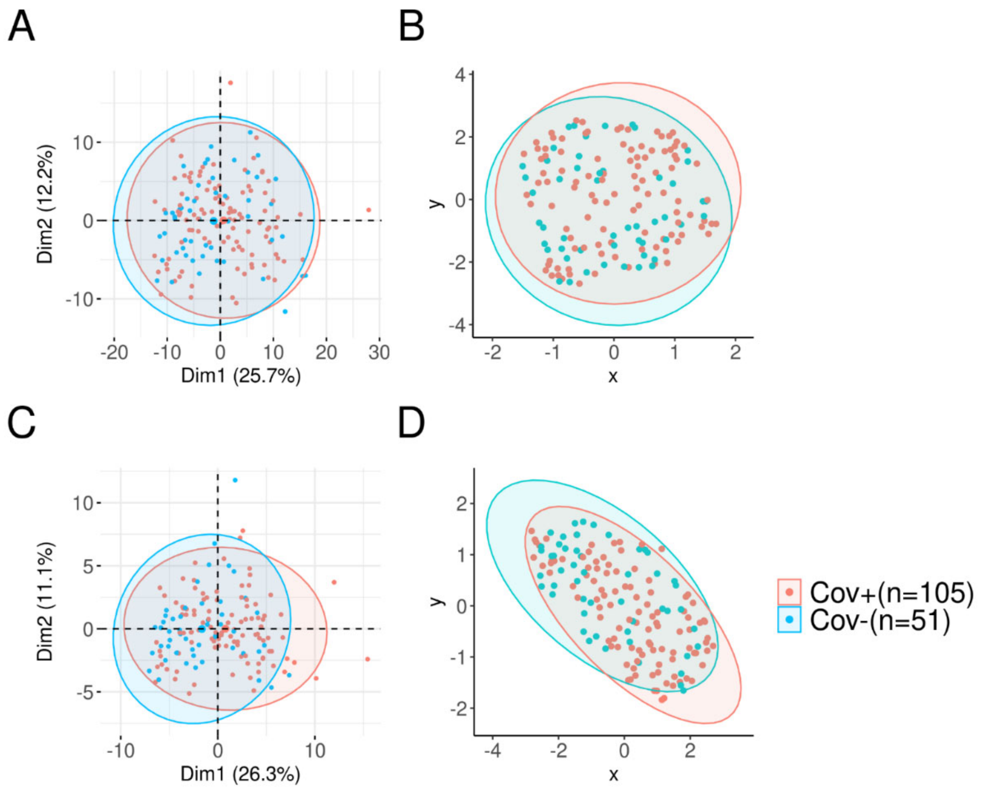

Figure S1. Reduction of dimension analysis performed from several sets of peaks for SARS-CoV-2(+) (n=105) and control (n=51). PCA **(A)** and UMAP **(B)** performed on all detected peaks. Ellipses define the boundary areas for the categorical variables projections PCA **(C)** and UMAP **(D)** performed on the 52 peaks selected with two-tailed Wilcoxon rank sum test ($p < 0.05$). PCA: Principal Component Analysis; UMAP: Uniform Manifold Approximation and Projection.
